# Motives for change of first-line antiretroviral therapy regimens in an unselected cohort of HIV/AIDS patients at a major referral centre in South-west Cameroon

**DOI:** 10.1186/s13104-017-2948-3

**Published:** 2017-11-28

**Authors:** Christian Akem Dimala, Ndemazie Nkafu Bechem, Desmond Aroke, Benjamin Momo Kadia

**Affiliations:** 1 Faculty of Epidemiology and Population Health, London School of Hygiene and Tropical Medicine, London, UK; 2 Orthopaedics Department, Southend University Hospital, Essex, UK; 3 Health and Human Development (2HD) Research Network, Douala, Cameroon; 4 Department of Public Health, University of Nottingham, Nottingham, UK; 5 Mbengwi District Hospital, Mbengwi, Cameroon; 6 Foumbot District Hospital, Foumbot, Cameroon; 7 Grace Community Health and Development Association (GRACHADA), Kumba, Cameroon

**Keywords:** Antiretroviral therapy, Cameroon, Regimen change

## Abstract

**Objective:**

The rapid scale-up of antiretroviral therapy (ART) coverage in sub-Saharan Africa has encountered the challenge of maintaining international clinical standards of ART utilization and change of ART regimens. In Cameroon, scarce reports have documented the motives for change of ART. This study had as objective to investigate the reasons for switch in ART through a secondary analysis and descriptive synthesis of data from a cross-sectional study at the Limbe Regional Hospital.

**Results:**

One hundred participants were included. Their mean age was 40.2 ± 8.0 years and 70% of them were females. The median duration of ART use was 60 months. Zidovudine-Lamivudine-Nevirapine regimen was received by 83% of patients while the Stavudine-Lamivudine-Nevirapine regimen had the highest median duration of use (58 months). Most patients had experienced changes in ART (especially from Stavudine-Lamivudine-Nevirapine regimen) with the chief reason being unavailability of their previous regimens. Four patients had their ART changed due to active tuberculosis, 4 due to pregnancy and 7 due to ART toxicity (4 and 3 for peripheral neuropathy and lipodystrophy respectively). In conclusion, shortages in ART hugely influenced switch in regimens. In such a context, modifications in ART possibly deviate from guidelines with resultant sub-optimal therapy and enhanced drug resistance.

**Electronic supplementary material:**

The online version of this article (10.1186/s13104-017-2948-3) contains supplementary material, which is available to authorized users.

## Introduction

Sub-Saharan Africa (SSA) with the highest burden of HIV/AIDS has witnessed enormous progress in antiretroviral therapy (ART) coverage programs, with a double-fold increase in ART coverage over the past 5 years [1]. As much as 12 million people were reported to be on ART in 2015, corresponding to an estimated 47% treatment coverage [1]. However, with this rapid scale-up of treatment coverage across SSA, it has been increasingly challenging to maintain the international clinical standards of ART roll-out and treatment protocol requirements within national HIV/AIDS control programs. In 2007, the Cameroon government took initiatives to scale-up the care for HIV/AIDS by providing antiretroviral drugs free of charge, just as in several other sub-Saharan African countries [2]. However, increasing reports suggest that changes in patients’ treatments do not fulfil clinical guidelines and unplanned treatment interruptions due to patient and healthcare supply-related factors are not uncommon [2]. Treatment failure and emerging resistance to ART agents among others are imminent consequences of these practices which tend to negatively impact overall treatment outcome. Much, therefore, remains to be done since the current coverage rates are sub-optimal, and the standards of patient management need to be upheld. A major step to achieving this is by studying the reasons underlying ART utilization and change in relation to the current standards of patient management in the midst of ART coverage scale-up. In Cameroon, scarce reports have documented the motives for ART changes among HIV/AIDS patients. The objective of this study was to investigate the reasons for switch in ART among HIV/AIDS patients on ART enrolled in a previous cross-sectional study conducted at the Limbe Regional Hospital in the South West region of Cameroon [3].

## Methods

### Study design and participants selection

This is a secondary analysis and descriptive synthesis of data from a cross-sectional study involving HIV/AIDS patients who visited the HIV treatment centre of the Limbe Regional Hospital in Cameroon. This hospital is a major referral centre in the South West Region of the country. Details of the participants selection, study procedures, data sources and measurements are described in the primary study [3]. This previous report had as aim to assess the association between ART and hypertension by comparing the prevalence of hypertension in HIV/AIDS patients on ART and ART-naïve patients. During the primary study period, CD4 cell count was done after diagnosing HIV seropositivity then 6 monthly for patients who were put on ART. Earlier CD4 cell counts could be requested in case of specific indications. For viral load testing, patients were referred to tertiary health care centres. A conclusion of treatment failure was mostly on the basis of immunological and clinical variables. We included patients aged 21 years and above and compliant to ART for at least 6 months.

### Study procedures and variables

Participants were enrolled via convenience sampling and were administered a questionnaire through a structured interview by the principal investigator to collect socio-demographic and medical data relevant to the study (Additional file 1).

### Data sources and measurements

The following parameters were collected from the participants through self-reports and from chart reviews: Age, sex, region of origin, marital status, employment status, medical history, opportunistic infection history, HIV infection history, ART use, duration of ART use and reasons for change of regimens.

### Data management, data analysis and synthesis

Data from the study was retrieved, cleared and analyzed using Epi-info version 7. Frequencies of the respective variables, means, standard deviations, medians and interquartile ranges were computed, and an overall descriptive synthesis of data was adopted as appropriate.

### Ethical considerations

Ethical approval for the primary study was obtained from the Institutional Review Board of the Faculty of Health Sciences, University of Buea.

## Results

### Socio-demographic and clinical characteristics of the participants

A hundred participants on ART were recruited. The mean age of the participants was 40.2 ± 8.0 years and 70% of them were female. Fifty-two percent of the participants were married and 69% practiced unskilled jobs (Table 1). Based on the WHO clinical staging of HIV infection, 52% of participants were at stage 3 of the disease against 5% at stage 1. The median duration of HIV infection and ART use were 68 and 60 months, respectively (Table 1).

**Table 1:** Socio-demographic and clinical characteristics of the participants

Characteristic	Study participants (n = 100)
Age (in years)	
Mean ± SD	40.2 ± 8.0
Range	26–64
Gender^a^	
Males	30 (30%)
Females	70 (70%)
Marital status^a^	
Married	52 (52%)
Unmarried	48 (48%)
Occupation^a^	
Skilled	12 (12%)
Unskilled	69 (69%)
Unemployed	19 (19%)
Region of origin^a^	
North west	46 (46%)
South west	41 (41%)
Others	13 (13%)
WHO clinical staging^a^	
Stage 1	5 (5%)
Stage 2	36 (36%)
Stage 3	52 (52%)
Stage 4	7 (7%)
Duration of HIV infection (months)	
Median (IQR)	68 (42–84)
Range	12–168
Duration of ART use (months)	
Median (IQR)	60 (36–72)
Range	12–132
CD4 cell count	
Median (IQR)	476 (361–619)
Range	49–977
Current opportunistic infections^a^	
Tuberculosis	1 (1%)
Previous opportunistic infections^a^	
Tuberculosis	20 (20%)
Herpes zoster	12 (12%)
Oral candidiasis	4 (4%)
Toxoplasmosis	1 (1%)

*IQR* interquartile range; *SD*: standard deviation

^a^Percentages in parenthesis correspond to the number of participants in the respective categories as a proportion of the total number of participants (n = 100)

A single participant had an opportunistic infection (tuberculosis) at the time of the study and 37 (37%) participants had past histories of one or more opportunistic infections with tuberculosis being the most frequent opportunistic infection (Table 1).

### Antiretroviral therapy regimens and drugs

All participants were on first-line antiretroviral therapy and the regimens included drugs of the nucleotide and non-nucleotide reverse transcriptase inhibitor classes. The various first-line regimens used as well as participants who had their regimens changed since initiation of ART are shown on Table 2. The regimen composed of Zidovudine-Lamivudine-Nevirapine was the regimen most received by the participants while that composed of Stavudine-Lamivudine-Nevirapine had the highest median duration of use (Table 2).

**Table 2:** Participants who used and had their regimens changed and the duration of use

Antiretroviral therapy regimens	Participants who ever received regimen	Participants who had their regimen changed (%)^a^	Median ART duration in months (IQR)	Range of ART duration in months
Zidovudine-lamivudine-nevirapine	83	43 (52.4%)	9 (7–14)	1–47
Tenofovir-lamivudine-nevirapine	65	20 (30.8%)	2 (2–3)	1–24
Stavudine-lamivudine-nevirapine	53	53 (100%)	58 (37–80)	8–110
Zidovudine-lamivudine-efavirenz	15	10 (66.7%)	13 (2–24)	1–69
Tenofovir-lamivudine-efavirenz	9	2 (22.2%)	9 (7–14)	1–47
Tenofovir-emcitrabine-nevirapine	2	0 (0.0%)	3 (2–4)	2–4
Tenofovir-emcitrabine-efavirenz	1	1 (100%)	29 (29–29)	29

^a^Percentages in parenthesis correspond to the number of participants who had their regimen changed as a proportion of the number of participants who ever received that regimen

Most of the participants had their ART regimens changed. The main reason for change of regimen was unavailability of the regimen they were previously on. Four participants had their regimens changed due to active tuberculosis infection and 4 had their regimens changed due to their pregnancy state. Seven participants had their regimens changed due to intolerable side-effects (4 and 3 for peripheral neuropathy and lipodystrophy, respectively). The various reasons for the change of ART regimens are summarized on Table 3.

**Table 3:** Reasons for change of anti-retroviral therapy regimens

Antiretroviral therapy regimens (number of participants who had regimen changed) and reasons for change of regimens	Number of participants who had their regimens changed per categories of the reasons of change (%)^a^
Zidovudine + lamivudine + nevirapine (n = 43)	
Stock out	43 (100%)
Tenofovir + lamivudine + nevirapine (n = 20)	
Stock out	20 (100%)
Stavudine + lamivudine + nevirapine (n = 53)	
Stock out	39 (73.6%)
Tuberculosis	4 (7.5%)
Peripheral neuropathy	4 (7.5%)
Lipodystrophy	3 (5.7%)
Pregnancy	3 (5.7%)
Zidovudine + lamivudine + efavirenz (n = 10)	
Stock out	10 (100%)
Tenofovir + lamivudine + efavirenz (n = 2)	
Stock out	1 (50%)
Anemia	1 (50%)
Tenofovir + emcitrabine + efavirenz (n = 1)	1
Stock out	1 (100%)

^a^Percentages in parenthesis correspond to the number of participants who had their regimen changed per specific reason of change, as a proportion of the total number of people who had that regimen changed

### Discussion

In SSA, changes in ART regimens were less reported before 2007 when antiretroviral drugs were expensive and less accessible. After 2007, switch in regimens became more frequent although in Cameroon information on this phenomenon, notably, the reasons for modifying regimens, has been grossly anecdotal. This study intended to explore the motives for change of ART regimens among HIV/AIDS patients receiving care at the Limbe regional hospital. It was observed that the Zidovudine-Lamivudine-Nevirapine regimen was most accessible while the Stavudine-Lamivudine-Nevirapine regimen had the longest median period of use. Most study participants had experienced changes in ART and this was mainly due to drug stock out. The Stavudine-lamivudine-Nevirapine formulation was the most changed regimen. Smaller proportions of patients who were initially on the above mentioned Stavudine-based regimen experienced changes in ART due to drug toxicity, tuberculosis (TB) co-infection and pregnancy. Anaemia caused a change in regimen in only one patient who was initially on Tenofovir-Lamivudine-Efavirenz.

In Cameroon, a previous study revealed that the prevalence of ever changing regimen at the Nkongsamba regional hospital was as high as 73.8% [4]. In this same study, over 90% of study participants had experienced an incidence of ART stock shortage [4] which was probably the chief reason for switch in regimens as in a previous report by Essomba et al. at the Edea regional hospital [5] and our study. These consistent findings suggest that the ART coverage programs in Cameroon may be flawed by inadequate strategies aimed at ensuring appropriate adjustments in ART supply in relation to increased demands. This is further evidenced by preliminary studies conducted out of Cameroon that rather advanced clinical reasons, in particular, drug toxicity as motives for switching ART regimens. Nonetheless, our findings concur with results of previous studies in that, drug toxicity, notably, peripheral neuropathy, was mainly noted among patients on Stavudine-based therapy [6–10]. Toxic effects of antiretroviral agents are often mild but occasionally, they may be serious enough to affect health and quality of life. Some of these may emerge months or years after initiation of treatment but others appear shortly after starting ART and disappear within a few weeks. Of note, peripheral neuropathy and lipodystrophy which could be secondary to reverse transcriptase inhibitors tend to worsen over time and may not regress. Thus, it may not seem surprising that the Stavudine-based formulation which had the longest duration of use among our study participants accounted for most cases of drug toxicity (peripheral neuropathy and lipodystrophy).

In 2010, the World Health Organization recommended the replacement of Stavudine by Tenofovir in treatment protocols due to its associated side effects. This change in guidelines, alongside drug stock outs, could be underlying reasons for the extensive change in Stavudine-based regimens observed after 2010. However, this information could not be accurately ascertained from individual patient records, possibly overestimating the extent of stock out of Stavudine-based regimens in our study. Whilst these revisions in the guidelines are plausible explanations for stock out in Stavudine-based regimens, changes in the regimens of patients containing this antiretroviral agent as a result of stock outs were observed among study participants prior to 2010.

Our findings also concur with previous studies in that TB was the only comorbidity that led to switch in regimens albeit previous studies reported much higher rates of TB co-infection [7, 11]. Among patients on regimens containing hepatotoxic drugs like Nevirapine (as noted among the TB infected patients in our study who were exclusively on Stavudine-Lamivudine-Nevirapine), co-infection with TB commands a change in such regimens because of the feared synergistic hepatotoxic effects of Nevirapine and anti-TB drugs. As regards pregnancy, switching to regimens with limited propensity for teratogenicity is a priority.

Prescribed ART regimens may be varied depending on specific circumstances like; contraindication of certain ART drug classes; ART drug interactions; ART drug resistance; severe intolerability of side effects and preferred regimens. Despite the extensive list of available protocols, patients are mainly on first-line regimens as reflected in our study population, with second line regimens being scarcely used, and third-line regimens are made available only in the treatment centers of tertiary hospitals. Viral load testing remains the preferred method of monitoring treatment failure. Recent WHO guidelines indicate that a repetitive viral load greater than 1000 copies/ml obtained on two consecutive measurements within a 3-month interval after at least 6 months of commencing new ART regimen is confirmatory of treatment failure. While CD4 and clinical monitoring may be informative in this regard, frequent changes in regimens on a background of limited access to viral load testing as in our setting could promote sub-optimal therapy and invariably, drug resistance. Switching can be done from one regimen of the first-line to another if indications for change of ART are met. Nonetheless, therapy is switched from first-line to second or third-line regimens mainly when there is development of resistance to first-line regimens. Third-line regimens should consist of new drugs with minimal risk of cross-resistance to previously used regimens and include integrase inhibitors, second generation non-nucleoside reverse transcriptase inhibitors and protease inhibitors [12].

In conclusion, shortages in ART hugely influenced switch in regimens in our study population. Policy makers and public health experts in Cameroon may need to anticipate on surges of newly diagnosed cases and make provisions for undiagnosed cases so as to limit mismatch in demand and supply of antiretroviral drugs. Meanwhile, drug toxicity and comorbidity remain pertinent causes of change of regimen. While modifications in ART may generally serve to maintain health, it should be ensured that the changes are done within the limits of guidelines in order to limit complications such as sub-optimal treatment regimens and drug resistance.

## Limitations

Shifts in regimens appeared to be prevalent in our setting but our study could not evaluate the possible impact on immunologic, virologic and clinical progress of the patients. Large scale prospective studies are clearly warranted in this regard. The small sample size possibly limits the array of potential reasons for switch in ART to those of the participants enrolled, as well as the generalizability of our findings. However, data on the reasons for change of ART regimens was collected as open-ended entries and therefore exhaustive within the confines of the study sample. The stock out of Stavudine-based regimens could be an overestimation since the replacement of Stavudine by Tenofovir in first line regimens as per the WHO recommendations after 2010, could not be adequately ascertained from the patients’ medical records. Recall bias during self-reports and incomplete data on chart reviews are other potential limitations of this study.

## Additional file

**Additional file 1.** Questionnaire: “The prevalence of hypertension in HIV/AIDS patients on antiretroviral therapy compared with art-naïve patients at the Limbe regional hospital”. In the primary study, a structured questionnaire was used to record sociodemographic, historical, clinical and laboratory variables per study participant.
