# Supplementary material for: Motives for change of first-line antiretroviral therapy regimens in an unselected cohort of HIV/AIDS patients at a major referral centre in South-west Cameroon
Source: BMC Res Notes. 2017 Nov 28;10:623. doi: 10.1186/s13104-017-2948-3 (PMC5704546; doi:10.1186/s13104-017-2948-3)
Supplement: Supplementary file 1 — Additional file 1. Questionnaire: “The prevalence of hypertension in HIV/AIDS patients on antiretroviral therapy compared with art-naïve patients at the Limbe regional hospital”. In the primary study, a structured questionnaire was used to record sociodemographic, historical, clinical and laboratory variables per study participant. [file 13104_2017_2948_MOESM1_ESM.doc]

**Supplementary File 1**

**QUESTIONNAIRE**

**THE PREVALENCE OF HYPERTENSION IN HIV/AIDS PATIENTS ON ANTIRETROVIRAL THERAPY**

**COMPARED WITH ART-NAÏVE PATIENTS AT THE LIMBE REGIONAL HOSPITAL**

**Main investigator:**

Date __________________

Section 1 – Identification and Past Medical History

| 1. | Identification (Centre code/Study code) |  |  |  |  |  |  |
| --- | --- | --- | --- | --- | --- | --- | --- |
|  |  |  |  |  |  |  |  |
| 2. | Age (in years) |  |  |  |  |  |  |
| 3. | Sex | 1. | Male |  | 2. Female |  |  |
| 4. | Occupation |  |  |  |  |  |  |
| 5. | Region of origin |  |  |  |  |  |  |
| 6. | Marital status | 1. | Married |  | 2. Unmarried |  |  |
|  |  | 3. Widow | |  |  |  |  |
| 7. | Medical history of any of the conditions beside | 1. Hypertension | | 2. Diabetes 3. Renal | | |  |
|  |  | failure 4. Stroke | |  | 5. None | 6. |  |
|  |  | Don’t know | |  |  |  |  |
| 8. | Time when diagnosis of above condition was | 1 - 1. < 12 months ago | | | 2. ≥ 12 months ago | |  |
|  | made | 2 - 1. < 12 months ago | | | 2. ≥ 12 months ago | |  |
|  |  | 3 - 1. < 12 months ago | | | 2. ≥ 12 months ago | |  |
|  |  | 4 - 1. < 12 months ago | | | 2. ≥ 12 months ago | |  |
|  |  | 5. | Don’t know |  |  |  |  |
| 9. | Treatment received for any of the above | 1. | Yes (Specify) |  | 2. None |  |  |
|  | conditions | 3. | Don’t know |  |  |  |  |
| 10. | A family history of hypertension | 1. | Yes |  | 2. No |  |  |
|  |  | 3. | Don’t know |  |  |  |  |
| 11. | If yes, for Family history of hypertension | 1. | 1^st^ degree relative (father, mother, brother, | | | |  |
|  |  | sister) | |  |  |  |  |
|  |  | 2. | 2^nd^ degree relative (grandparents, uncle, | | | |  |
|  |  | aunt, cousins) | |  |  |  |  |
| 12. | Smoking | 1. Yes | |  | 2. Yes but stopped | |  |
|  | (Tobacco product – cigarettes, cigar, pipes) | 3. | No |  |  |  |  |
| 13. | Duration of smoking (If yes or yes but | 1. | < 12 months |  | 2. ≥ 12 months | |  |
|  | stopped) |  |  |  |  |  |  |
| 14. | Quantification of smoking (If yes/yes but | 1. | ≤ 10 sticks/day |  | 2. 10-20 sticks/day | |  |
|  | stopped) | 3. | ≥ 20 sticks/day |  |  |  |  |
| 15. | Alcohol consumption | 1. Yes | |  | 2. Yes but stopped | |  |
|  |  | 3. | No |  |  |  |  |
| 16. | Duration of alcohol cons (If yes/yes but | 1. | < 12 months |  | 2. ≥ 12 months | |  |
|  | stopped) |  |  |  |  |  |  |

| 17. | Quantification of alcohol consumption (If | | 1. | | ≤ 15 units/week (6 bottles/week) | | | | | |  |  |
| --- | --- | --- | --- | --- | --- | --- | --- | --- | --- | --- | --- | --- |
|  | yes/yes but stopped) (1 local beer bottle = 2.5 | | 2. | | 15 – 20 units/week (6-8 bottles/week) | | | | | |  |  |
|  | units) | | 3. | | > 20 units/week (8 bottles/week) | | | | | |  |  |
| 18. | Physical activity/exercise | | 1. | | Yes |  |  |  | 2. Yes but stopped | |  |  |
|  |  |  | 3. | | No |  |  |  |  |  |  |  |
| 19. | Quantification of physical exercise | | 1. | | < 3 hours brisk walking/week | | | | | |  |  |
|  |  |  | 2. ≥ 3 hours brisk walking/week | | | | | | | |  |  |
| 20. | Date of diagnosis of HIV (day/month/year) | |  |  |  |  |  |  |  |  |  |  |
| 21. | Duration since the diagnosis of HIV (in | |  |  |  |  |  |  |  |  |  |  |
|  | months) | |  |  |  |  |  |  |  |  |  |  |
| 22. | Date of start of ART (day/month/year) | |  |  |  |  |  |  |  |  |  |  |
| 23. | Duration since the start of ART (in months) | |  |  |  |  |  |  |  |  |  |  |
| 24. | Adherence to ART (Number of times when ART | | 1. | | None |  |  |  |  |  |  |  |
|  | was missed in the past six months) | | 2. | | ≤ 1 week (7 dozes) in the past 6 months | | | | | |  |  |
|  |  |  | 3. | | 1-2 weeks (7-14 dozes) in the past 6 | | | | | |  |  |
|  |  |  | months | | |  |  |  |  |  |  |  |
|  |  |  | 4. | | 3-4 weeks (15-29 dozes) in the past 6 | | | | | |  |  |
|  |  |  | months | | |  |  |  |  |  |  |  |
|  |  |  | 5. | | > 4 weeks (30 dozes) in the past 6 months | | | | | |  |  |
|  |  |  | 6. | | Don’t know | |  |  |  |  |  |  |
| 25. | Any opportunistic infection diagnosed since | | 1. | | Tuberculosis | |  | 2. Candidiasis | | |  |  |
|  | diagnosis of HIV status | | 3. | | Toxoplasmosis | |  | 4. PCP | | |  |  |
|  |  |  | 5. | | Herpes Zoster | |  | 6. Wasting syndrome | | |  |  |
|  |  |  | 7. | | Others (specify) | |  | 8. None | | |  |  |
| 26. | If opportunistic infection, treatment received | | 1. | | Yes (Specify) | |  | 2. None | | |  |  |
|  |  |  | 3. | | Don’t know | |  |  |  |  |  |  |
| 27. | Duration of treatment of above mentioned | | 1. | | < 12 months | | 2. ≥ 12 months | | | |  |  |
|  | infection | |  |  |  |  |  |  |  |  |  |  |
| 28. | Any other drug received for ≥ 1 month | | 1. | | Oral Contraceptive Pills | | | | 2. Steroids | |  |  |
|  | (since you were diagnosed of being HIV + ) | | 3. | | Sympathomimetics | |  |  | 4. NSAIDS | |  |  |
|  |  |  | 5. | | Others (specify) | |  |  | 6. None | |  |  |
| 29. | Any other major health problem | | 1. | | Yes (Specify) | |  | 2. No | | |  |  |
|  |  |  | 3. | | Don’t know | |  |  |  |  |  |  |
| **Section 2 – Follow Up data/Retrospective data** | | |  |  |  |  |  |  |  |  |  |  |
| 30. | **ART-treated:** Blood pressure before ART start | |  | 1. Hypertension ( | | |  | mmHg) | | | |  |
|  | **ART-naïve:** Blood pressure at HIV diagnosis | |  | 2. No hypertension ( | | |  |  | mmHg) | | |  |
|  |  |  | |  | |  |  |  | |  | |  |
| 31. | **ART-treated:** Parameters before ART | Weight (Kg) | | | | Height |  | BMI (Kg/m^2^) | | CD4 count | |  |
|  | start |  |  |  |  | (cm) |  |  |  |  |  |  |
|  | **ART-naïve:** parameters at HIV diagnosis |  |  |  |  |  |  |  |  |  |  |  |
|  |  |  |  |  |  |  |  |  |  |  |  |  |

**Section 3 - Physical exam**

1. Weight (kg)
2. Height (cm)
3. Body mass index (kg/m^2^)
4. Waist circumference (cm)

| 36. | Hip circumference (cm) |  |  |  |  |
| --- | --- | --- | --- | --- | --- |
| 37. | Waist-Hip ratio |  |  |  |  |
| 38. | First Blood pressure reading (mmHg) |  |  |  |  |
| 39. | Second Blood pressure reading (mmHg) |  |  |  |  |
| 40. | Mean blood pressure (mmHg) |  |  |  |  |
| 41. | WHO Clinical Staging of HIV | 1. Stage 1 | 2. Stage 2 | 3. Stage 3 | 4. |
|  |  | Stage 4 |  |  |  |
| 42. | Opportunistic infection |  |  |  |  |

**Section 4 – Paraclinicals (where applicable)**

1. Recent blood cholesterol level
2. CD4 cell count (in the past 3 months)
3. Others (specify)

**LIST OF ANTIRETROVIRAL DRUGS Duration of use of Antiretroviral drug:**

**A. Individual antiretroviral drugs**

| **Antiretroviral drug (trade name)** | | | **Yes/No** | **Duration of** | **Comments and reasons for** |
| --- | --- | --- | --- | --- | --- |
|  |  |  |  | **use** | **change** |
| 1. | Abacavir, (Ziagen) | |  |  |  |
| 2. | Zidovudine,(ZDV) | |  |  |  |
| 3. | Stavudine, (Zerit) | |  |  |  |
| 4. | Lamivudine, (Epivir) | |  |  |  |
| 5. | Didanosine,(Videx) | |  |  |  |
| 6. | Tenofovir, (Viread) | |  |  |  |
| 7. | Emtricitabine (Emtriva) | |  |  |  |
| 8. | Nevirapine, (viramune, nevimune) | |  |  |  |
| 9. | Zalcitabine | |  |  |  |
| 10. | | Efavirenz, (Stocrin, efavir) |  |  |  |
| 11. | | Delavirdine, (rescriptor) |  |  |  |
| 12. | | Lopinavir/ritonavir, (Aluvia) |  |  |  |
| 13. | | Indinavir, (Crixivan) |  |  |  |
| 14. | | Nelfinavir, (viracept) |  |  |  |
| 15. | | Fosamprenavir |  |  |  |
| 16. | | Saquinavir, (Fortorase, invirase) |  |  |  |
| 17. | | Atazanavir |  |  |  |
| 18. | | Ritonavir, (Norvir) |  |  |  |
| 19. | | Amprenavir, (agenerase) |  |  |  |

**B. Antiretroviral therapy regimens**

| **Antiretroviral drug combination (Trade name)** | | **Yes/No** | **Duration of** | **Comments and** |
| --- | --- | --- | --- | --- |
|  |  |  | **use** | **reasons for change** |
| **2 NRTI + 1 NNRTI** | |  |  |  |
| 1. | Zidovudine + Lamivudine + Efavirenz, |  |  |  |
| (Duovir/ combivir/Zidolam + stocrin) | |  |  |  |
| 2. | Zidovudine + Lamivudine + Efavirenz |  |  |  |
| 3. | Tenofovir + Lamivudine + Efavirenz |  |  |  |
| 4. | Tenofovir + Lamivudine + Nevirapine |  |  |  |
| 5. | Tenofovir + Emtricitabine + Efavirenz |  |  |  |
| 6. | Tenofovir + Emtricitabine + Nevirapine |  |  |  |
| (Atripla) | |  |  |  |
| 7. | Lamivudine + Stavudine + Nevirapine |  |  |  |
| (Triommune/Nevilast) | |  |  |  |
| 8. | Others |  |  |  |
| **2 NRTI + 1 PI/r** | |  |  |  |
| 1. | Tenofovir + Lamivudine + |  |  |  |
| Lopinavir/ritonavir | |  |  |  |
| 2. | Tenofovir + Lamivudine + |  |  |  |
| Atazanavir/ritonavir | |  |  |  |
| 3. | Zidovudine + Lamivudine + |  |  |  |
| Lopinavir/ritonavir | |  |  |  |
| 4. | Zidovudine+ Lamivudine + |  |  |  |
| Atazanavir/ritonavir | |  |  |  |
| 5. | Zidovudine + emtricitabine + |  |  |  |
| Lopinavir/ritonav | |  |  |  |
| 6. | Abacavir + Didanosine + |  |  |  |
| Lopinavir/ritonavir | |  |  |  |
| **3** | **NRTI** |  |  |  |
| 1. | Abacavir + lamivudine + zidovudine |  |  |  |
| (Trizivir) | |  |  |  |
| 2. | Tenofovir + Lamivudine + Zidovudine |  |  |  |
